# Assigning F-words as ingredients of interventions for children with cerebral palsy functioning at GMFCS IV and V: A scoping review protocol

**DOI:** 10.3389/fresc.2023.1110552

**Published:** 2023-02-16

**Authors:** E. Longo, R. Monteiro, Á. Hidalgo-Robles, G. Paleg, C. Shrader, A. C. De Campos

**Affiliations:** ^1^ Department of Physical Therapy, Federal University of Paraíba, João Pessoa, PB, Brazil; ^2^ Department of Physical Therapy, Federal University of São Carlos, São Carlos, SP, Brazil; ^3^ Facultad de Educación, Universidad Internacional de La Rioja, Logroño, La Rioja, Spain; ^4^ Montgomery County Infants and Toddlers Program, Rockville, MD, USA; ^5^ HMS School for Children with Cerebral Palsy, Philadelphia, PA, USA

**Keywords:** children, cerebral palsy, early intervention, F-words, ICF, non-ambulant

## Abstract

**Introduction:**

Children with Cerebral Palsy (CP) functioning at Gross Motor Function Classification System (GMFCS) levels IV and V require “on time” identification and intervention. Interventions offered continue to be a challenge, in high-, but even more so in middle-, and low-income countries.

**Aim:**

To describe the methods developed to explore the ingredients of published studies on early interventions in young children with cerebral palsy (CP) at highest risk of being non-ambulant based on the “F-words for child development framework” and the design of a scoping review exploring these ingredients.

**Method:**

An operational procedure was developed through expert panels to identify ingredients of published interventions and related F-words. After sufficient agreement among researchers was reached, a scoping review was designed. The review is registered in the Open Science Framework database. The “Population, Concept and Context” framework was used. Population: young children (0–5 years with CP and at highest risk for being non-ambulant (GMFCS levels IV or V); Concept: non-surgical and non-pharmacological early intervention services measuring outcomes from any ICF domain; Context: studies published from 2001 to 2021. After duplicated screening and selection, data will be extracted and quality will be assessed with the American Academy for Cerebral Palsy and Developmental Medicine (AACPDM) and Mixed Methods Appraisal (MMAT) tools.

**Results:**

We present the protocol to identify the explicit (directly measured outcomes and respective ICF domains) and implicit (intervention features not explicitly intended or measured) ingredients.

**Conclusion:**

Findings will support the implementation of the F-words in interventions for young children with non-ambulant CP.

## 1. Introduction

In recent years, much progress has been made in the care of children with cerebral palsy (CP). The availability of robust screening tools has made it possible to provide early identification and referral for infants with CP. Using Prechtl's General Movement Assessment (GMA) and Hammersmith Infant Neurological Examination (HINE), we are now able to identify which babies may be at risk for a non-ambulant prognosis of gross motor function, i.e., those who are likely to be classified at levels IV and V of the Gross Motor Function Classification System (GMFCS), as early as 2–9 months of corrected age (1).

Children with non-ambulant motor delays and impairments in CP have also been shown to reach 90% of their gross motor potential by age 3 years (2). This does not mean, however, that there is no room for change in several areas of functioning. Indeed, recent studies have indicated that early, family-centered, and context-focused interventions may favor several areas of development for young children with CP (3). These findings are still to be applied to young children at risk of being non-ambulant. This population has the right to receive on time intervention (i.e., offering motor experience and participation at the same time as typically developing children at their chronological adjusted age) that is comprehensive and suited to their needs.

The framework of the International Classification of Functioning, Disability and Health (ICF) (4) has largely contributed to the understanding of the multiple elements that are relevant to health. These must be addressed when carrying out interventions to increase “participation”, reducing functional limitations and associated disabilities. More recently, the F-words in childhood development framework (5) has helped to raise awareness about how we should think, speak, and act when caring for children with CP: focusing on Functioning (Activity), Family (Environmental Factors), Fitness (Body Structure and Function), Fun (Personal Factors), Friends (Participation), and Future. Research has shown this framework has been increasingly used to support a holistic approach to childhood disability around the world (6). However, it is unclear to what extent these concepts are incorporated when considering young children with CP and at highest risk for being non-ambulant.

Having CP and functioning at GMFCS levels IV and V increases the risk of having associated comorbidities (e.g., seizures, visual impairment, eating disorders, among others) (7) and thus the range of interventions children may benefit from. Despite efforts to increase the evidence base for early interventions, particularly motor interventions (8, 9), no comprehensive reviews of interventions addressing the range of health-relevant outcomes for children who are at highest risk to be non-ambulant with CP were encountered. Of particular interest is understanding how the complex health needs of young children with this condition are addressed today. Equally important is the well-being of their parents because we know now how closely these outcomes are linked (10).

Identifying the active ingredients of interventions (i.e., what makes it work, including elements such as intervention dosage, principles, etc.), is key to facilitating the implementation (11). As the F-words may not be explicitly addressed in studies, it is therefore important to reveal ingredients associated with F-words, so that future recommendations can be made on how to design interventions that promote the F-words for children with CP. This is especially relevant for those who are non-ambulant, as often they do not receive evidence-based interventions (12).

This protocol paper will describe the methods developed for data extraction and analysis and the search methods for a scoping review that will explore the ingredients of early interventions in young children with cerebral palsy (CP) at highest risk to be non-ambulant and identify the ICF components and F-words addressed by the interventions. With the results, it is expected to provide a general menu of interventions offered to the population of interest, their strengths, and limitations, and to make recommendations for future research framed within the ICF and the F-words.

## 2. Methods

The methods described in this manuscript for identification of intervention ingredients were developed in a preparatory stage for a scoping review. This scoping review will be conducted using the “Population, Concept and Context” (PCC) framework that is recommended for scoping reviews (13).

The PCC framework is more appropriate than the PICO tool for capturing the wide range of study designs associated with the sub-questions, as in this review, quantitative, qualitative, and mixed methods studies and reviews are all relevant.

This protocol was registered at OSF registries, under the DOI: 10.17605/OSF.IO/RXY9Z.

### 2.1. Study design

This scoping review will be based on the recommendations (14) and its execution will follow five steps: (1) description of the research questions; (2) identification of relevant studies; (3) selection of studies; (4) data mapping; and (5) summary and reporting of results. The review will be reported in accordance with the Preferred Reporting Items for Systematic Reviews and Meta-Analyses extension for Scoping Reviews (PRISMA -ScR) guidelines.

#### 2.1.1. Step 1: identification of the research question

The overarching research questions that will guide this scoping review are:

1. Which interventions are available for young children with CP at GMFCS levels IV and V and their families?
2. What are the ingredients of these interventions?
3. Which ICF/F-Words domains are included in these interventions?

#### 2.1.2. Step 2: identification of relevant studies—search strategy

During this stage, a duplicated process will be used in the process of screening articles for inclusion in the study. The search strategy was developed by the 4 researchers of the team. They discussed and agreed extensively on inclusion and exclusion criteria.

The literature search will take place through electronic databases, using structured terms applicable to individual databases, which will include combinations and variations of the keywords: and the agreement with Boolean operators AND/OR.

Systematic searches will be performed in the following databases: Pubmed, Web of Science, Cinahl and Scopus, for studies published in the last 20 years (2001–2021) in English, Portuguese or Spanish. The keywords will include “cerebral palsy”, “quadriplegia”, “spastic cerebral palsy”, “infants”, “children”, “early intervention”, “rehabilitation”, “enriched environment”.

The search strategy will be tested to verify its suitability for the selected databases and keywords.

#### 2.1.3. Step 3: study selection

This study will use the PCC (Population, Concept and Context) framework, proposed by the Joanna Briggs Institute, to guide the search strategy, as described in Table 1 (15).

**Table 1:** PCC definitions.

PCC elements	Definition (according to JBI Reviewer Manual, Chapter 11)
Population	Infants or children aged 0 to 5 years with CP at high risk to function at GMFCS IV or V (at least 30% of the sample)
Concept	Interventions available for young children with CP who are non-ambulant and their families classified according to the ICF domains and linked to F-words, considering the active, direct and indirect ingredients.
Context	High-, middle- and low-income countries All settings considered Original research articles, regardless of design Published in English, Portuguese and Spanish

The stages of the study will be followed through the inclusion and exclusion criteria, described below.

##### 2.1.3.1. Inclusion criteria

Study sample: infants or children aged 0–5 years with CP in GMFCS IV or V (at least 30% of the sample). For studies that address participants younger than 2 years, we will consider other motor scoring tools in addition to the GMFCS [e.g., GMA Motor Optimality Scores, HINE, Gross Motor Function Measure (GMFM) curves] when available to estimate severity (16).

Study designs: RCTs, clinical studies (other than RCTs), qualitative studies and mixed-methods studies, if they describe either interventions or outcomes related to interventions (e.g., experiences of families with therapy, access to services, referral processes).

Outcome areas: any outcomes related to activity, participation, body function and structures, and contextual factors.

##### 2.1.3.2. Exclusion criteria

Surgical, pharmacological or any other invasive interventions.

Study sample (<30% under 5 years of age, non-CP, GMFCS I-III, animal studies.

Study design: study protocols, methods papers, outcomes not related to children (e.g., back pain in caregivers).

Two independent reviewers will make the initial selection and conflicts will be resolved by consensus with a third reviewer.

#### 2.1.4. Step 4: description of the preparation process to map the data

To develop the methods for this study, the authors had meetings with experts in the field to discuss the approach to F-Words mapping. Next, there were several discussions within the group for test rounds of the process, to standardize the level of agreement within the team. Initially, 7 randomly selected studies were discussed and the first version of the document was created that guide the identification of intervention ingredients and related F-words (with instructions on how to identify the F-words of interventions/results). An independent review of 10 research-based randomized studies was then performed, which assessed which ingredients fell under each F-word. Finally, an independent analysis of 3 additional studies based on the document was carried out. Researchers reached 70% agreement, which was considered sufficient for the purposes of this study.

Next, the authors completed the process of listing components of early interventions, which will later be used in the scoping review to describe the explicit and implicit ingredients.

The explicit ingredients, defined as those directly measured (Table 2) will be identified through the assessment instruments used in the studies. A database of ICF components encompassed by the measurement tools of each study was mapped based on available resources^1^ or published studies. When information was not found, ICF components were identified by expert opinion. Whenever the measure targeted the family/therapists and not the child, we assigned Environment as the ICF component. The next step consisted of linking the ICF domains to the respective F-words.

**Table 2:** Explicit ingredients of of the interventions for children with CP GMFCS levels IV and V.

F-word	Explicit ingredients (examples of measures)
Family 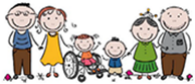	Use of measures including parents’ perceptions (of children, process, routines, functional domains, etc.) through study-specific measures or standardized tools (e.g., MPOC-56). Satisfaction (e.g., MRPS) and expectations were assessed mainly through specific study measures. Family-related outcomes such as empowerment, sense of support or priorities were directly assessed (e.g. FES, FSS, CPCHILD), as well as family stress, burden of care and other related outcomes (e.g. Parenting stress index, Caregiver Strain Index, NOSI-K, PSSNICU, DASS-21, MCSI, PSI-4-SF, SRQ20, and other study-specific measures). Family Impact Measure and other impact measures (e.g. FIATS, IFS, PedsQLTM-Family Impact Module) were included. Study-specific measures were used to assess parents’ knowledge of CP and other family-related outcomes [e.g. BPAQ, HOME, AHEMD-IS, impact of environmental modification, PEDI (Caregiver Assistance domain), Judson Scale].
Functioning 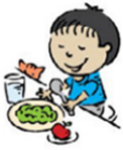	Use of outcome measures that directly address the Activity domain (multidomain assessments, e.g., BSID-III, DAYC, PEDI/ PEDI-CAT, WeeFIM, Batelle), Box and Block Test, reaching test. Mobility was assessed mainly through standardized measures (e.g. GMFM-88, GMFM-66, AIMS, TIMP, IMP, PDMS-2, BMFM, MFM, FMFM, Sitting Assessment Scale, FMS5, FMS50, FMS500). Other power mobility measures included WSC and ALP. Self-care domain (e.g., Modified Functional Feeding Assessment, GAS for functional goals) and social, language, and behavioral development (e.g., Rosetti Infant-Toddler Language Scale, PLS-4, UPAS, ECI) were direct ingredients. Other function related outcomes included problem solving in play (APSP) and study specific measures (e.g., home activity log).
Fitness 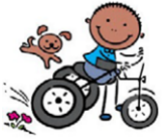	Use of outcome measures that directly address the body structures and function domain (e.g. weight, height, arm circumference, skinfold, chest health, etc.). Motor and neurological function measures included GMA, HINE, TINE, GDS, or reflex integrity. Neuromusculoskeletal and movement-related functions included measures as ROM, House Classification of hand function, TCMS, Sitting Assessment Scale, 6MWT, number of reciprocal steps, walking speed and endurance, EMG. Muscle tone function measures were also included (e.g.Tardieu Scale, MAS, ALT muscle tone scale), as well as postural oscillations and ECAB were used to evaluate sensory functions. Bones structures were assessed through MP and other x-ray measurements, DEXA, bone ultrasound, BMD. Likewise, posture and joint positions (e.g., Oxford Assessment Tool for Complex Disability, clinical examination of contractures). Outcomes for voice and speech function included study specific measures, and standardized tools (e.g. MLUm, Questionnaire for Dysarthria of Puyuelo). Clinical dysphagia symptoms were measured with Pedi-Eat-10, and FEES. Other fitness-related outcomes included BSFS, CSHQ-AF, or TGF-b1 concentration.
Future 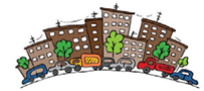	Long-term outcomes include classification systems (GMFCS, MACS, EDACS, CFCS), BSID-III, Batelle, CSHQ-AF, ROM, etc. Registries and study specific measures were important in “future” (qualitative reports of attitudes, access to services, CP epidemiology, etc.)
Friends 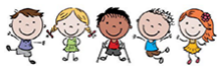	Use of outcome measures that directly address participation, including areas of play, skill development, physical recreation, and social activities (e.g. COPM, APCP, CEDL, ITQoL, GAS for participation goals, FIATS).
Fun 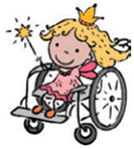	Measures of enjoyment, reports of happiness, contentment and leisure were included within “fun” F-word. Examples of measures were home activity log interview, PEDI-CAT, FIATS, and GAS or COPM for leisure goals.
Other Factors 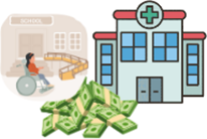	Additional measures included CP registries (CP epidemiology, access to services, etc.), medical records, and service provision (questionnaires on intervention, MPOC-56, parents and professionals’ expectations for rehabilitation). Socioeconomic measures were frequently assessed (e.g. Household Form, Poverty Measurement Tool, nutritional intake, etc.). Other F-words related outcomes included parents’ knowledge of CP.

6MWT, 6-Minute Walk Test; AHEMD-IS, Affordances in the Home Environment for Motor Development- Infant Scale; AIMS, Alberta Infant Motor Scale; ALP, Assessment of Learning Power mobility use; ALT, Arms, Legs and Trunk muscle tone scale; APCP, Assessment of Preschool Children's Participation; APSP, Assessment of Problem Solving in Play; BMD, Bone Mineral Density; BMFM, Bimanual Fine Motor Function; BSFS, Bristol Stool Form Scale; BPAQ, Buss-Perry Aggression Questionnaire; CEDL, Child Engagement in Daily Life questionnaire; CFCS, Communication Function Classification System; COPM, Canadian Occupational Performance Measure; CPCHILD, Caregiver Priorities and Child Health Index of Life with Disabilities Questionnaire; CSHQ-AF, Children's Sleep Habits Questionnaire-Abbreviated Form; DASS-21, Depression, Anxiety and Stress Scale; DAYC, Development Assessment of Young Children Evaluation Tool; DEXA, Dual-energy x-ray absorptiometry; ECAB, Early Clinical Assessment of Balance; ECI, Early Coping Inventory; EDACS, Eating and Drinking Ability Classification System; FEES, Fiberoptic Endoscopic Evaluation of Swallowing; FES, Family Empowerment Scale; FIATS, Family Impact of Assistive Technology Scale; FMS, Functional Mobility Scale; FMFM, Fine Motor Function Measure; FSS, Family Support Scale; GAS, Goal Attainment Scaling; GDS, Gesell Development Scale; GMA, Prechtl General Movements Assessment; GMFCS, Gross Motor Function Classification System; GMFM-66, Gross Motor Function Measure 66-item; GMFM-88, Gross Motor Function Measure 88-item; HINE, Hammersmith Infant Neurological Examination; HOME, Home Observation Measurement of the Environment—infant-toddler version; IFS, Impact on Family Scale; IMP, Infant Motor Profile; ITQOL, Infant and Toddler Quality of Life Questionnaire; MACS, Manual Ability Classification System; MAS, Modified Ashworth Scale; MCSI, Modified Caregiver Strain Index; MFM, Motor Function Measure questionnaire; MLUm, Mean Length of Utterance in morphemes; MP, Measurement of Migration Percentage; MPOC-56, Measure of Processes of Care; MRPS, Medrisk Instrument for Measuring Patient Satisfaction with Physical Therapy Care; NOSI-K, Nijmeegse Ouderlijke Stress Index questionnaire, short version; PEDI, Pediatric Evaluation of Disability Inventory; PEDI-CAT, Pediatric Evaluation of Disability Inventory Computer Adaptive Test; Pedi-Eat-10, Pediatric Eating Assessment Tool-10; PedsQLTM-FIM, Paediatric Quality of Life-Family Impact Module; PDMS-2, Peabody Developmental Motor Scales; PLS-4, Preschool Language Scale-Fourth Edition; PSI-4-SF, Parenting Stress Index-Short Form; PSSNICU, Parental Stress Scale for NICU patients; ROM, Range Of Motion; SRQ20, Self-Reporting Questionnaire 20 items; TCMS, Trunk Control Measurement Scale; TGF-b1, Growth factor b1; TIMP, Test of Infant Motor Performance; TINE, Touwen Infant Neurological Examination; UPAS, Uniform Performance Assessment System; WeeFIM, Functional Independence Measure for Children; WSC, Wheelchair Skills Checklist.

**Figure 1:**
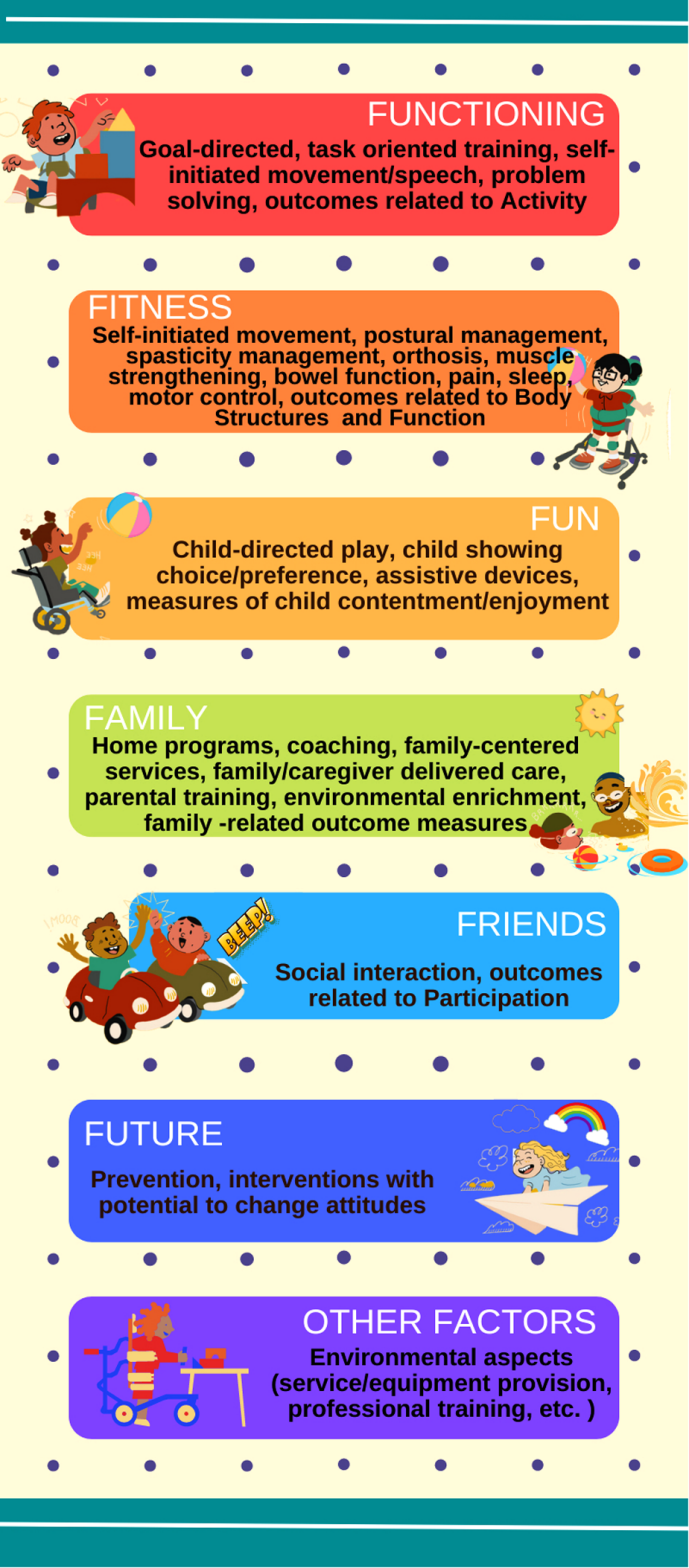
Implicit ingredients of the interventions.

To identify implicit ingredients (Figure 1), defined as features of the intervention not explicitly intended or measured, the following criteria were established: (1) An identified intervention unit was defined as an ingredient of the approach, and this intervention unit that composes each ingredient was closely linked to each F-word. For example, the ingredient “Family-Centered Services” will be linked to the “F-word” “family”. All the ingredients of the included interventions will be identified and linked to each F-word based on the consensus by 4 authors. At all stages of analysis, interventions that do not fit into any of the F-words will be described in a group called “other Factors”.

#### 2.1.5. Step 5: map the data

The steps for mapping the data will be:

(1) A form will be used by the research team, *via* Google forms, to map the data, including variables such as Study title; First Author; Year; Country of origin. Significant POPULATION: Total number included in study (include drop outs and controls); Are 30% GMFCS Level IV and V; Are 30% age 0–5 (below age 6); If less than 30% GMFCS IV/V or under 6 years, explain reason for inclusion; Describe population and any groups or subgroups; Number of children completing study—if different for subjects and controls please describe; Were any typical children included—describe; Description of subjects e.g. diagnosis, GMFCS levels, functional abilities; Number of controls (if appropriate); Description of controls (if appropriate); Age range and mean (SD); Number and ages of GMFCS IV if able to separate; Number and ages of GMFCS V if able to separate; If there were two groups—were the similar study groups. ICF Domains covered by outcome measures; F-words addressed (if any); If survey—whose opinion was sought?; Range Of Motion (ROM) results (overall—both significant and trends); Muscle tone results; Muscle strength results; Gastro-intestinal results; Cardiovascular results; Mental functions results; Skin functions results; Pain results; Tolerance of device/time or program results; Quality of life results; Activities of Daily Living results; Gross engine activity results; Fine motor activity results; Play or communication results; Care burden or costs results; Contextual factors results; parent satisfaction; Occupational performance results; Patient oriented measures results; Describe intervention—or what the study measured; Describe control intervention (if delivered).
(2) Two pairs of reviewers will extract data from randomly assigned articles and rate the quality of the study using the Mixed Methods Appraisal tool (MMAT) (16). The selection of the tool was due to the heterogeneity of the designs of the studies that will be included in the review. The MMAT is a critical assessment tool that promotes an analysis of the methodological quality of systematic reviews of mixed studies categorized into: qualitative research, randomized controlled studies, non-randomized studies, quantitative descriptive studies and mixed methods studies. It is recommended that at least two independent reviewers are involved in this process, and that they have experience or training in the domains to be evaluated. The results of the quality of the studies will be computed through the number of items covered in relation to the total number of items.
(3) Reviewers will score the MMAT of each article and questions will be discussed in remote meetings of the research team.

#### 2.1.6. Step 6: gather, summarize, and report the results

This research seeks to gather data to advance knowledge on intervention for infants or children aged 0–5 years with CP in GMFCS IV or V to attract attention to children at highest risk of being non-ambulant, through mapping of interventions that are being delivered to this public, or aspects relevant to these interventions. The intervention's ingredients or themes will be linked to the F-words.^2^ (https://www.canchild.ca/en/research-in-practice/f-words-in-childhood-disability).

The objective is to identify interventions according to the F-words: Family, Functioning, Fitness, Future, Friends and Fun, and to define what each F-word represents and covers in terms of intervention ingredients.

The PCC inclusion criteria will guide the data map. Therefore, at least three tables will be created for data entry. The first will describe the number of countries where data were collected for each study. The second will summarize the characteristics of the included studies and the third will describe the ingredients of the interventions related to the components of the ICF and F-words. A descriptive summary will accompany the tabulated results and describe how the results apply to our scoping review questions.

## 3. Results and discussion

This scoping review will contribute to filling an important knowledge gap, concerning the population of children with CP and GMFCS levels IV and V, who are the least likely to receive evidence-based interventions. Figure 1 describes examples of implicit ingredients of interventions based on the consensus step among authors.

On Table 2, we present the mapping of outcome measures and the corresponding ICF domains/F-words. They were considered “explict” ingredients of the interventions.

The decision to describe the ingredients as either “explicit” or “implicit” was due to the observation that many studies utilize a “bottom-up” approach, i.e., address separate components of a skill in the intervention, and measure outcomes that may not be directly related to those components. This finding has been previously reported in a scoping review investigating studies targeting participation through a focus on body structures and functions (17). Our strategy to overcome this issue was to map the explicit (measured) outcomes, as well as the implicit (i.e., intervention components that may not have been measured) for a comprehensive understanding of what is implied in the interventions mapped. This information will be useful to guide the design of future interventions and research using the F-words.

It was often difficult to clearly define which ingredients were involved in interventions due to the lack of details on what was done. In some studies, it was not possible to map ingredients due to descriptions such as “standard therapy”, which is not sufficient information to understand the components of the intervention. Another example was “play-based approach”, which did not clearly meet the criterion for “Fun” as defined in our protocol (i.e., child-directed play). Nevertheless, our approach showed sufficient agreement among researchers and was considered satisfactory for use in the planned scoping review.

Additional challenges in describing intervention ingredients and related F-words in the present study were related to the fact that the population of interest includes young children who have limited motor repertoire.

For this reason, specific aspects needed to be defined. For example, very young children often do not interact directly with same-age peers. We therefore included as ingredients related to Friends any social interactions, including with adult family members and other adults, such as teachers.

Also, taking into consideration that self-initiated movement is challenging for children functioning at GMFCS levels IV and V, we related to Fitness any small movements they could initiate to get some level of physical activity, even if using assistive devices. The same was valid for Functioning, where self-initiated activities included adapted ones. Self-initiated movement is a shared ingredient belonging to both Fitness and Functioning, as the understanding of the study team was that it may affect both areas.

Family is the F-word chosen to represent the ICF component of Environment, as it is a fundamental contextual factor of children. In this protocol, interventions engaging the family at any level (e.g., family-delivered interventions, family-centered care, etc.) and all measures of family-related outcomes (e.g., parent stress, family quality of life, etc.) were related to this word. We acknowledge that there are several other relevant environmental factors playing an important role in the functionality and health of young children, such as service provision, professional training, and attitudes. These were mapped under “Other factors”. Although the word “factors” also starts with an F, we do not suggest that this should be a new F-word, as the framework is now well established and starts to promote significant impact in the field in its current format (6).

Not related to any of the ICF components, the F-word Future was originally conceived as a reminder of the constantly “becoming” nature of childhood (5). Especially when considering children at GMFCS levels IV and V, there are several prevention actions to be taken in anticipation of their risks for musculoskeletal conditions such as hip displacement, scoliosis, and other complications that may ultimately lead to pain and loss of quality of life (18). This was taken into consideration when mapping preventing-related ingredients to this F-word, however, considerable overlap may occur with ingredients related to Fitness, which will cause the same component of the intervention to be assigned to both F-words. Additionally, interventions that may have the potential to alter the course of CP (either in a positive or negative direction) could also be considered for this F-word. Finally, thinking about transition to adulthood starts early, or at least it should, as this is a major concern of families from the moment they receive a diagnosis (19). We therefore included under this F-word interventions that aim to provide knowledge on the condition for families, or to change how society sees children with disabilities as potential strategies to alter the life course of children.

We described here an innovative approach to assign ingredients of interventions, and acknowledge that it has potential limitations. However, the methodological rigor in the process of identifying the explicit and implicit ingredients ensured an adequate level of agreement between the evaluators. In the future, we recommend the use of the International Classification of Health Interventions (ICHI), recently published by the WHO (20), as a common tool for reporting the interventions aimed at young children with CP classified as GMFCS levels IV and V.

## 4. Patient and public involvement

One of the team members directly involved in developing the protocol is an occupational therapist student who has life experience in CP. Claire Shrader is a triplet and her two brothers have CP (GMFCS level III and IV). She attended and experienced several early intervention programs during her childhood and follows the needs of young adults with CP and advocates, together with her family, for the rights of people with CP. She is now an Occupational Therapist working with children at a school who are mostly non-ambulant, her mother and brother are employed as advocates, her father is an orthopedic surgeon specializing in CP and her other brother is studying for his PhD is Archeology focusing on disability history.

## Data Availability

The raw data supporting the conclusions of this article will be made available by the authors, without undue reservation.

## Footnotes

^1^https://www.sralab.org/rehabilitation-measures; https://pediatricapta.org/fact-sheets/.

^2^https://www.canchild.ca/en/research-in-practice/f-words-in-childhood-disability.

## 6. Conclusions

Identifying the direct and indirect ingredients of the available interventions according to the F-words may help researchers, professionals and families of young children with non-ambulant CP reflecting on the importance of considering all relevant outcomes for early stimulation programs and ensuring improved future studies. We invite researchers to use this methodology so in the future more robust studies such as meta-analyses can be conducted providing evidence on interventions using the F-words.

## Strengths and limitations of this study

Identifying intervention ingredients is key to promote implementation of evidence-informed practices.

• To the best of our knowledge, this study will be the first to review different approaches to interventions for GMFCS IV and V children, exploring their active ingredients and correlating them with the F-words.
• The results are expected to be useful to stakeholders in designing and implementing rehabilitation interventions that cover all F-words.
• By developing consensus on this methodology, it can be used by others to make meta-analyses easier.
